# Neural Activity in the Macaque Putamen Associated with Saccades and Behavioral Outcome

**DOI:** 10.1371/journal.pone.0051596

**Published:** 2012-12-10

**Authors:** Jessica M. Phillips, Stefan Everling

**Affiliations:** 1 Graduate Program in Neuroscience, Western University, London, Ontario, Canada; 2 Department of Physiology and Pharmacology, Western University, London, Ontario, Canada; 3 Brain and Mind Institute, Western University, London, Ontario, Canada; 4 Robarts Research Institute, London, Ontario, Canada; Barrow Neurological Institute, United States of America

## Abstract

It is now widely accepted that the basal ganglia nuclei form segregated, parallel loops with neocortical areas. The prevalent view is that the putamen is part of the motor loop, which receives inputs from sensorimotor areas, whereas the caudate, which receives inputs from frontal cortical eye fields and projects via the substantia nigra pars reticulata to the superior colliculus, belongs to the oculomotor loop. Tracer studies in monkeys and functional neuroimaging studies in human subjects, however, also suggest a potential role for the putamen in oculomotor control. To investigate the role of the putamen in saccadic eye movements, we recorded single neuron activity in the caudal putamen of two rhesus monkeys while they alternated between short blocks of pro- and anti-saccades. In each trial, the instruction cue was provided after the onset of the peripheral stimulus, thus the monkeys could either generate an immediate response to the stimulus based on the internal representation of the rule from the previous trial, or alternatively, could await the visual rule-instruction cue to guide their saccadic response. We found that a subset of putamen neurons showed saccade-related activity, that the preparatory mode (internally- versus externally-cued) influenced the expression of task-selectivity in roughly one third of the task-modulated neurons, and further that a large proportion of neurons encoded the outcome of the saccade. These results suggest that the caudal putamen may be part of the neural network for goal-directed saccades, wherein the monitoring of saccadic eye movements, context and performance feedback may be processed together to ensure optimal behavioural performance and outcomes are achieved during ongoing behaviour.

## Introduction

A major advancement in our understanding of basal ganglia (BG) function has been the concept of largely segregated cortico-BG circuits subserving motor, oculomotor, executive, and limbic functions [1]. According to this prominent model, most cortical regions send topographic projections through various striatal, pallidal and thalamic zones, and thalamo-cortical projections return these circuits to frontal cortical sub-regions. The putamen mainly receives inputs from sensorimotor cortices and projects back to the same motor areas through the lateral globus pallidus and ventrolateral and ventro-anterior nuclei of the thalamus (VA/VL), forming the “sensorimotor circuit” [2]. The caudate nucleus belongs to the “associative” and “oculomotor” loops, and controls eye movements with inputs from the dorsolateral prefrontal cortex, and the frontal and supplementary eye fields (FEF and SEF) [2]. Phasic caudate activation inhibits the substantia nigra pars reticulata (SNpr), thereby releasing the superior colliculus from tonic inhibition just prior to a saccade [3].

Likely influenced by this prominent model, electrophysiological studies in monkeys have almost exclusively examined the functional properties of putamen neurons during reaching and grasping tasks, while investigations of the striatal contribution to saccade behavior have focused on the caudate. This concept of effector-specialization for the caudate and putamen has been challenged by an increasing number of functional neuroimaging studies in human subjects, which have reported activation not only in the caudate nucleus but also, or sometimes exclusively in the putamen during saccadic eye movement tasks [4], [5], [6], [7], [8], [9], [10], [11], [12]. Some of these studies have supported a role for the putamen in the performance of anti-saccades, which requires the suppression of a saccade towards a flashed peripheral stimulus (pro-saccade) in favour of a saccade toward the opposite direction, compared with the performance of pro-saccades [13]. Neggers et al. (2012) have recently hypothesized that the apparent effector-specialization within the monkey striatum may be the result of an early bias in the field [14], [15], or alternatively that the saccade circuitry might be fundamentally different between humans and monkeys [4]. Although the latter alternative is possible, recent investigations of human white matter pathways have demonstrated that cortico-BG projection patterns are remarkably similar to the homologous pathways previously revealed by tracer studies in monkeys [4], [16], [17]. Further, comparative functional neuroimaging studies have demonstrated, at least in cortical areas, a large degree of homology between humans and macaques [18], [19]. In fact, macaque monkeys also show greater activations in the putamen during the performance of anti-saccades compared with pro-saccades [20]. Most importantly, macaque tracer studies demonstrate that caudal putamen neurons receive projections from the FEF and SEF [21], [22], [23], [24].

To directly investigate the role of caudal putamen neurons in goal-directed saccades, we conducted extracellular neuronal recordings in this region of the striatum while rhesus macaques performed alternating blocks of pro- and anti-saccades. We found that roughly half of the putamen neurons in the population were responsive to one or more features of the oculomotor task, which argues against the notion of effector-specialization in the caudate and putamen of primates, and is in agreement with human functional neuroimaging studies.

## Materials and Methods

### Subjects and Ethics Statement

Two male macaque monkeys (*Macaca mulatta,* 9.8 and 8.6 kg), monkey A and monkey B, were trained on a pro-/anti-saccade paradigm. All experimental methods described were conducted according to the guidelines of the Canadian Council on Animal Care policy on the care and use of experimental animals, and an ethics protocol (2008-125) approved by the Animal Users Subcommittee of the University of Western Ontario Council on Animal Care. Animals were pair-housed unrestrained in primate cages (Primate Products Inc.). Environmental enrichment was provided by dedicated animal husbandry staff in the form of gnawing wood, toys, perches, foraging boards, and other environmental items that could be manipulated by the animals.

### Implant and Surgery

In preparation for chronic electrophysiological experiments, each monkey underwent a surgical procedure in which a head restraint post and a plastic recording chamber were implanted. Animals were premedicated with atropine (0.05 mg/kg IM), and bupreonorphine (0.03 mg/kg IM), then induced with ketamine (10–15 mg/kg IM). The animals were then intubated and an IV catheter was placed in the cephalic vein of one arm and the saphenous veins of both legs. IV lactated ringers or normosol was provided at a maintenance rate. General anesthesia was maintained through IV propofol (0.3–0.4 mg/kg/min IV), and isoflurane gas (as required but usually 1%) delivered in nitrous oxide (0.5 l/min) and oxygen (2.0 l/min). Heart rate, blood pressure, O_2_ saturation, respiratory rate, ET CO_2_ and body temperature were recorded every 5–10 min for the duration of the surgery. A midline incision was made through the skin over the cranium. A plastic recording chamber was placed over a 19-mm diameter craniotomy situated above the premotor cortex of the right hemisphere at an angle that permitted access to the caudal putamen (36° and 38° Monkey A and Monkey B, respectively). Cranial implants were held in place with dental acrylic (methyl methacrylate) and secured to the skull with ceramic bone screws (Thomas Recording, Inc., Giessen, Germany). A plastic head post to restrain the head during the experiments was positioned stereotaxically and then secured to the skull will dental acrylic, which bonded together all the screws to form a single robust implant. The wound edge was cleaned and 1 to 3 absorbable sutures inserted (4-0 vicryl in sample interrupted pattern) to keep the skin closely apposed to the implant until healing had occurred. Animals were monitored by a veterinary technician until they recovered from anesthesia and could sit up in the cage. To alleviate any post operative discomfort, animals received analgesics buprenorphine (0.01–0.03 mg/kg tid) and meloxicam (0.2 mg/kg initially then 0.01 mg/kg) for the first 48 hours and then as required. Analgesics were given during induction and again as the animals began to recover from anesthesia. Animals were inspected at least twice a day by a trained staff member for the first three days post surgery. Animals were given 1–2 weeks to recover before training began. The monkeys were under close supervision by the university veterinarians for the duration of the study.

### Behavioral Task

**Figure 1:**
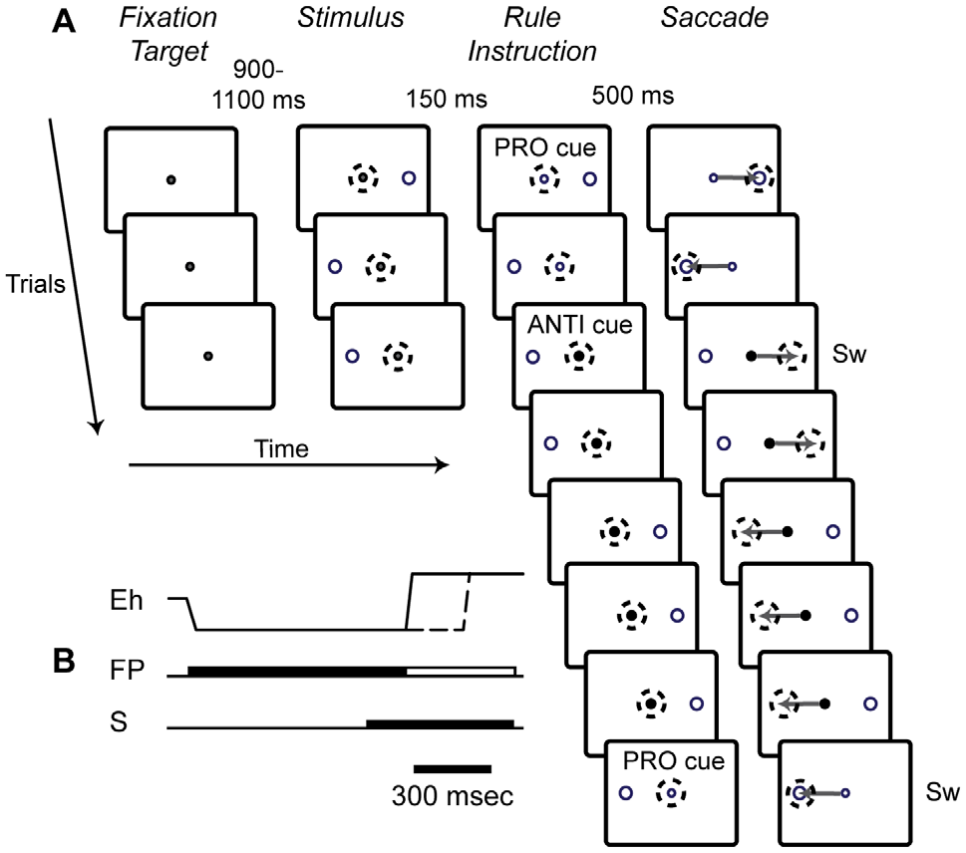
Behavioral task. In this task, the monkeys had to continuously alternate between blocks of pro- and anti-saccade (PRO cue, ANTI cue) trials. Responses under these two rules have opposite stimulus-response associations. A. This figure shows a schematic of the visual stimuli, with correct monkey behavior, for a possible trial sequence in the saccade-overriding task (SOT). The first trial in the sequence is represented by the top row in the schematic. The task was presented in blocks, in which the rule was constant. The rule switched after 5 to 10 correct trials were performed. Dashed circles represent the gaze location of the monkey, and the gray arrows represent the saccade trajectory toward the appropriate response window. Sw indicates that a row represents a switch trial. B. The temporal sequence of visual stimuli, including fixation point (FP), stimulus (S) and horizontal eye position (Eh) for an example trial. The time, at which the fixation point changes to an instruction cue, is indicated by the unfilled portion of the image that represents the FP. This trial shows behavior corresponding to a fast mode saccade (Eh, solid line) for which the rule and corresponding task-set was maintained internally from the previous trial, since the Eh changes almost as soon as the instruction cue comes on. Overlaid in this same figure is example behavior corresponding to a slow mode saccade (Eh, dashed line) for which the visual instruction cue was used to implement the task-set for the saccadic response.

The monkeys were trained on a pro-/anti-saccade paradigm, which was a modified version of the “saccade-overriding task”(SOT) introduced by Isoda and Hikosaka to investigate both automatically prepared and controlled saccade responses [25], [26] (Fig. 1). We chose this particular task design because in addition to a possible role in pro- and anti-saccade performance, previous studies have provided evidence that the putamen is involved in task-set reconfiguration [27], [28] and also that the contextual demands for top-down control can differentially engage the rostral versus the caudal striatum [29], [30], [31], [32], [33], [34], [35], [36]. In this task, the animals had to complete short, variable length blocks of a particular stimulus-response mapping rule. The two rules were (1) pro-saccade (saccade toward the peripherally presented stimulus) and (2) anti-saccade (suppress the pro-saccade response and instead, look toward the diametrically opposite location). The rule was constant within a block and switched when the block was completed. The length of each block was varied randomly between 5 and 10 trials with correct responses. During each trial, the monkey had to acquire a central, white fixation point. Next, after a random delay of 900 to 1100 msec, a green stimulus was presented to either the left or right of the fixation point at 8° eccentricity. After a fixed delay of 150 msec, the white fixation point was replaced with a coloured instruction cue, which indicated the rule of the current block. A green cue indicated the pro-saccade rule, while a red cue indicated the anti-saccade rule. If the correct response was generated, and the saccade endpoint fell within the appropriate target window (5°×5°) within 500 msec and was maintained for 80 msec, the monkey received a liquid reward between 0 and 500 msec later. The animals were not rewarded if the response was initiated before the fixation point changed colour. The monkeys performed between 4 and 232 task switches, with an average of 60 switches, per recording session.

### Single-neuron Recording Procedures

After each monkey was implanted with a plastic recording chamber, anatomical MRIs (T2 weighted) were obtained to aid in the guidance of electrode trajectories using a 7 Tesla Varian scanner. At the beginning of each recording session, a single tungsten microelectrode (UEWLFELMNN1E, FHC Inst., Bowdoinham, Maine) was lowered, using a manually controlled hydraulic microdrive (Narishige, Japan), through a semi-chronically implanted 23 gauge stainless steel guide-tube positioned approximately 5 mm above the dorso-lateral surface of the caudal putamen. The dura was pierced using a sterile spinal needle-tip and the guide tube was lowered through a grid that was fixed inside the recording chamber. The guide tube could be moved within the chamber in 1 mm steps using the x-y coordinates of the grid (15 mm diameter). Neural activity was amplified and bandpass filtered (150 Hz to 8 kHz), and single neurons were isolated using principle component analysis in the Plexon SortClient software package (Plexon Inc., Dallas, TX). We recorded the activity of all well-isolated neurons that were encountered without a pre-screening procedure. Horizontal and vertical eye positions were recorded using video eye-tracking at 500 Hz (EyeLink II, SR Research, Kanata, ON). The behavioral task and reward delivery were controlled by the CORTEX experimental control system (NIMH, Bethesda, MD), and all task events and digitized neural signals were stored together using the Plexon MAP system (Plexon Inc., Dallas, TX).

### Data Analysis

All analyses were performed offline using custom-written software in Matlab (Mathworks, Natick, MA). Saccade onset was defined as the time at which the radial eye velocity exceeded 30°/sec, and the time of the endpoint was considered to be that at which this parameter fell below this value. Accurate saccade onset and offset trial and categorization by CORTEX was verified by visually examining the eye traces from each session and manually correcting or removing any erroneously categorized saccades.

Our first question was whether or not the putamen neurons displayed task-related activity during the saccade period. To explore the time-course of potential task-related modulation in the entire population, we administered a two-way ANOVA with the factors task rule (pro, anti) and saccade direction (ipsiversive, contraversive) on successive 100-msec analysis epochs. These epochs began 500 msec preceding saccade onset and were sampled successively until 500 msec following saccade onset. We also repeated the same analysis but here we also included the percentage of “generally responsive” neurons at each epoch. These neurons were classified in this way if, over all conditions, saccade epoch activity minus baseline values were significantly different from zero (p<0.01). We used the results of these analysis to guide the following step.

Next, we measured task-related responses in a 100-msec window centered at saccade onset, from which baseline activity, sampled during a period in which the monkeys were fixating (100 msec following the acquisition of central fixation for 100 msec), was subtracted. We then entered these values into a two-way ANOVA with factors task rule (pro, anti) and saccade direction (contraversive, ipsiversive) to classify the neurons as task-related during the saccade period (p<0.01). The neurons were also classified as “generally responsive” if, over all conditions, saccade epoch activity minus baseline values were significantly different from zero (p<0.01) as described above. We included these neurons in the remaining analyses of the saccade period.

To visualize the population neural activity, we plotted the mean population activity as peri-event spike time histograms (PSTH), which were convolved using an asymmetric kernel that models a post-synaptic potential with a time constant of 20 msec [37] [37]. This was chosen over a Gaussian and other options as it ensured that any observed deviations from baseline activity would not be misinterpreted to occur at a time earlier than that which occurred in the recorded neuronal signal.

We also employed a receiver-operating characteristic (ROC) analysis to statistically compare neural activity distributions between trials categories having comparisons of interest. Specifically, a 100 msec-wide sliding window, in successive 1-msec steps, was used to calculate the time-course of the mean ROC area for activity distributions of comparative interest for each neuron, and used these values to calculate and plot the time-course of the mean ROC area for the population of neurons. To obtain 95% confidence intervals to denote the level of ROC area at which a significant difference between the two activity distributions occurred, we applied a sliding-window (1-msec steps) bootstrapping procedure [38].

**Figure 2:**
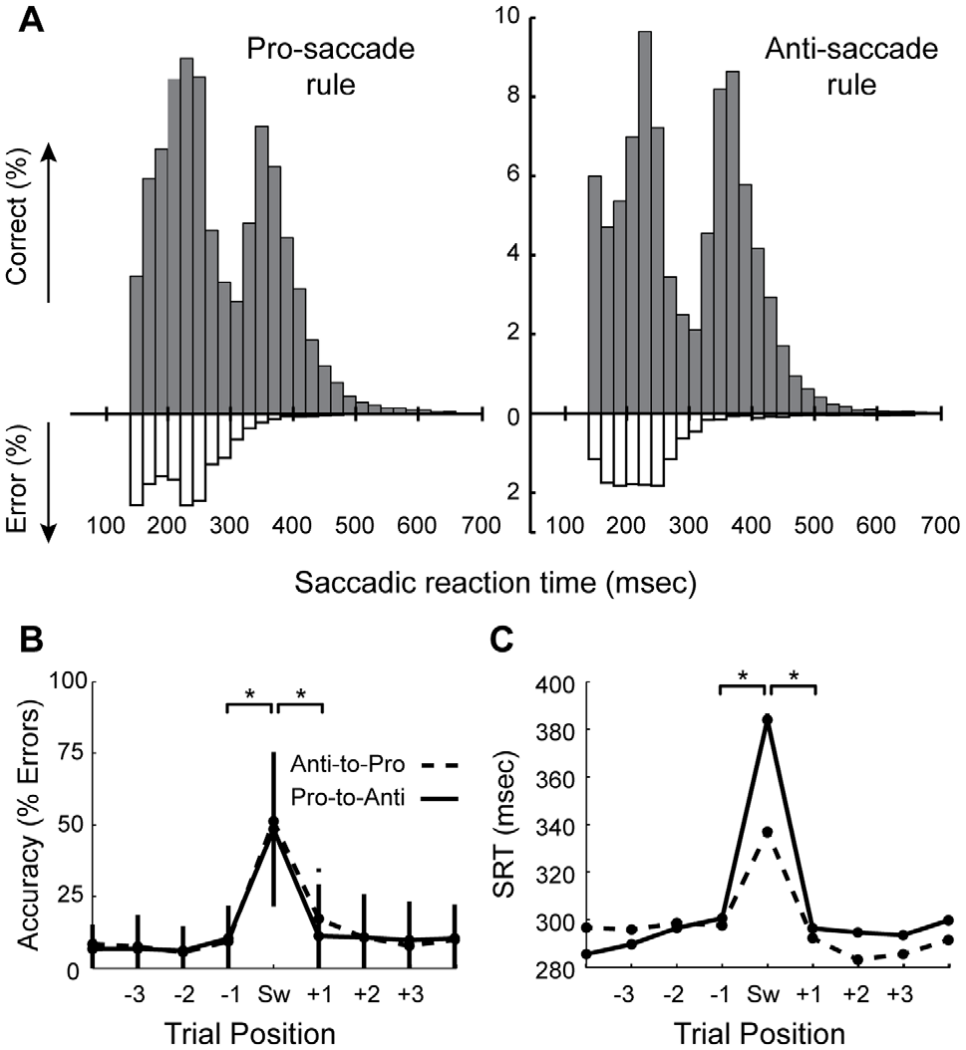
Saccade overriding task Performance. A. Saccadic reaction time (SRT) distributions for pro- and anti-saccade responses. Under each rule, a bi-modal distribution was obtained, which indicates that the monkeys used two different strategies to prepare their responses. Specifically, the animals used two different methods for rule application, which were intertwined with their speed-accuracy trade-off. Error trials were mostly generated under the internally-cued fast preparatory mode, and very rarely if at all under the slower controlled preparatory mode. B. Performance by trial-position, with respect to the switch trial. C. SRT by trial-position, with respect to the switch trial. In B&C, statistically significant switch costs are evident (* p<0.001, Wilcoxon ranked sum test). Switch costs are defined as a drop in performance on a switch trial, relative to repeat trials. On average, the correctly performed rule-switch trials were performed at latencies above 300 msec, which indicates that the monkeys had to prepare for the saccade using the slower controlled mode in order to successfully perform a rule-switch trial. Performance dropped to 50% on average (for both rules) on switch trials, which indicates that for approximately half of these trials, the monkeys prepared their response automatically based upon the rule in the previous trial, which should always lead to an error when faced with a switch trial.

The next question was whether or not activity in single putamen neurons was influenced by the mode used to prepare the saccade (short-latency internally cued versus long-latency externally cued) in each trial. To estimate the cutoff saccade reaction times (SRTs) between the distributions for fast- and slow-response trials, we inspected SRT histograms (Fig. 2A), separately for each rule, and chose a bisection point closest to the center between the two modal peaks. We then used this to separate the trials into groups based upon the mode of response preparation for subsequent statistical analyses.

A salient feature of many putamen neurons, which was apparent during recording sessions, appeared to be modulation by trial outcome (correct/rewarded vs. error/unrewarded). Therefore, we aimed to quantify the population activity in relation to the outcome of the just-executed response. To probe the time-course of these outcome-related modulations, we again measured activity in successive, 100-msec analysis epochs, beginning 500 msec prior to saccade onset for 1500 msec, and subjected the activity of each neuron to a three-way ANOVA. This time, the factors were task rule (pro, anti), saccade direction (contraversive, ipsiversive) and trial outcome (correct, error). Based on these results, we next chose a 200-msec wide analysis window beginning 200 msec following the saccade, to classify the putamen neurons that were sensitive to trial outcome.

### Reconstruction of Recording Locations

We made an effort to selectively target the anatomical regions within the caudal putamen that correspond to regions of cortical eye field inputs in previous tracer studies [21], [22], [23], [24]. To estimate the location of the single neurons from which we recorded, we used the anatomical MRIs together with coronal slices taken from the BrainInfo Template Atlas (histological drawings; National Primate Research Center, University of Washington Seattle, WA, http://www.braininfo.org). We fitted the appropriate anterior/posterior coronal slices onto the anatomical MRI for each animal. We then used the grid placed inside the chamber during MRI scans as a guide for the anterior-posterior and medial-lateral coordinates within the recording chamber. The location of each recorded neuron was placed on the composite MRI/map image. Next, we traced the putamen from the composite map to isolate this brain structure. We used the results from the statistical tests of single neuron activity to classify a neuron as saccade, task or outcome modulated (or a combination of these factors).

## Results

### Behavior

In the SOT, the rule was held constant within each block, and the peripheral stimulus occurred prior to the instruction cue. This task design provided the monkeys with a choice between two options: (1) to use the spatial location of the visual stimulus to prepare the response prior to the onset of the instruction cue, in anticipation that the task rule (pro or anti) would remain unchanged, a preparatory strategy that prioritized speed over accuracy which enabled short SRT responses, or (2) to release the task-set after the trial or to withhold an automatically planned saccade, and await for the instruction cue to apply the appropriate stimulus-response mapping rule, a preparatory strategy that prioritized accuracy over speed (and was associated with longer SRTs due to the stimulus-instruction asynchrony). Thus, the task allowed the animals to perform goal-directed saccades using two modes of preparation that differed in speed and accuracy priority levels. In the remainder of this article, we use the term *mode* to differentiate these fast and slow responses. The constant delay between peripheral stimulus presentation and instruction cue onset (which also served as a nonspecific go signal on short SRT trials) allowed the animals to execute a fast saccade based on the maintained task-set while the unpredictable length of the short constant-rule blocks encouraged the cautious, accurate mode of responding. The animals had to maintain a balance between speed and accuracy prioritization in their preparatory strategies to perform optimally.

In Figure 2, we have plotted the behavioral data. Histograms of SRTs for all completed trials for the two task rules are shown in Figure 2A. These plots show that the SRT distributions are not uni-modal, which stands in contrast with SRT distributions that have previously been obtained for other pro- and anti-saccade task variants in monkeys [13], [39], [40]. We verified that SRT the distributions for correct trials under both the pro- and anti-saccade rule were statistically bi-modal (p<0.0001, Hartigan’s dip test). These results indicate that in a proportion of the trials, saccades were quickly initiated which we assume to have been prepared *automatically* during trials in which the monkeys favoured speed. For these short-SRT trials, the monkeys had to maintain the task-set from the previous trial, and use it together with the spatial location of the stimulus to prepare their response in advance of instruction cue onset. The notion of automaticity here is taken to reflect faster processing times. We assumed then, that for the remaining proportion of trials, which was associated with longer SRTs, the monkeys prepared their responses in a *controlled* manner. For these responses, the monkeys would have to await the instruction cue so that it could be used to implement the stimulus-response mapping rule for saccade generation. Further, the fact that responses associated with these longer latencies were much more accurate than those prepared using the automatic mode (see Figure 2A) indicates that in this controlled mode, the animals favoured accuracy. As such, this behavior appears to reflect two different preparatory task-processing modes, which can be differentiated by the associated speed-accuracy prioiritization.

### Putamen Neurons are Modulated during the Peri-saccade epoch

**Figure 3:**
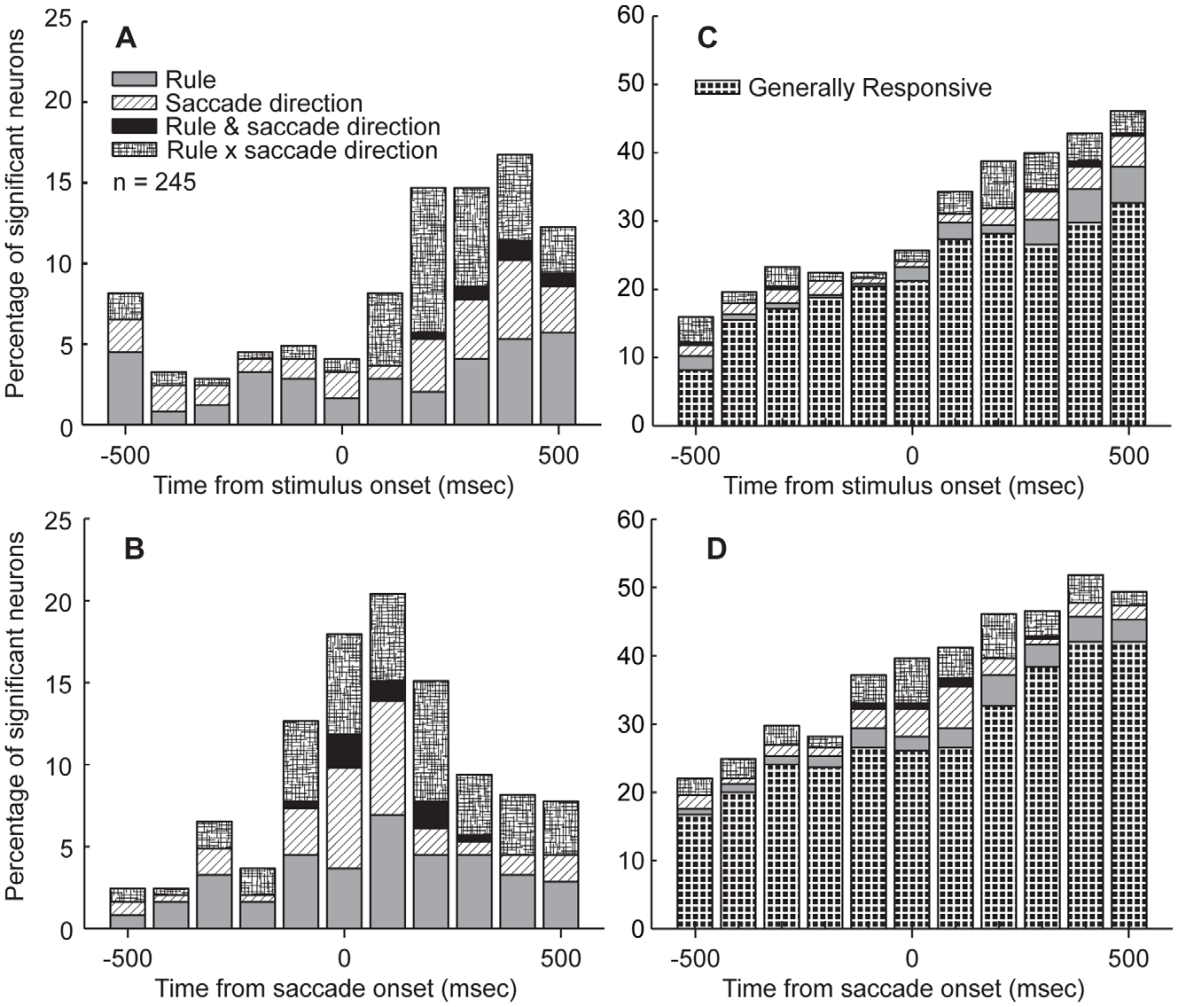
Time-course of ANOVA-identified percentage of task-modulated neurons. In order to explore the time-course of task-selectivity in the population of putamen neurons, we employed a two-way ANOVA with factors rule (pro, anti) and saccade direction (contraversive, ipsiversive) to examine putamen neuron activity in progressive 100-msec analysis bins around the peri-stimulus (A) and peri-saccade (B) period. The sharper and larger peak in the percentage of modulated neurons prompted us to use a peri-saccade analysis window for the remaining analyses of task-related modulations. C&D. We plotted the same time-courses of population selectivity again, but included the percentage of “generally responsive” neurons, which are characterized as having analysis epoch activity that is significantly different from zero when baseline values are subtracted (C shows peri-stimulus time-course and D shows the peri-saccade time-course).

We recorded the activity of 245 single neurons in the putamen (122 from monkey A and 123 from monkey B) while the monkeys performed the SOT. We employed a two-way ANOVA on successive 100-msec analysis epochs to explore the time-course of task-related modulations in the population of recorded neurons (p<0.01). The results of the ANOVA, for each analysis epoch, were used to place each neuron into one of four (Figure 3A,B) or one of five mutually-exclusive categories (Figure 3C,D) that indicated a main effect of either one or two factors, or an interaction between the two factors (Rule x saccade direction). For example, if the activity of a neuron showed a main effect of rule but also a rule x saccade direction interaction, we ignored the main effect and placed it in an interaction category. This approach ensured that each neuron was only counted once. The results are plotted in Figure 3. In Figure 3A, the time-course of task-modulations is aligned to the peripheral stimulus presentation. This figure demonstrates that task-modulations appeared after stimulus onset and indicates that putamen neurons did not tend to show preparatory task-related modulations. In Figure 3B, the time-course of task-modulations are aligned to the onset of the saccade, and here a peak in the percentage of significant neurons in the population is evident beginning with the bin centered on 100 msec prior to saccade onset, persisting into the bin centered at 200 msec following saccade onset, after which the percentage of significantly modulated neurons in the population decays toward pre-saccadic levels. Thus, the population of putamen neurons appeared to show a larger degree of task-modulation during the saccade period compared to the preparatory epoch of the trial. This conclusion is supported by the magnitude of the peak percentage modulated in each figure; for stimulus-aligned time-course the maximum percentage modulated neurons was roughly 16% whereas for the saccade-aligned time-course, the maximum was approximately 21%. In Figures 3C&D, the same time-courses of neuronal modulations in the population are plotted again but in these plots, the generally responsive neurons are included. These figures indicate a sizeable percentage of generally responsive neurons, and that this percentage grows toward the saccade epoch and then continues to grow beyond this epoch. We interpreted this feature to indicate a possible responsiveness to the outcome of the trials (a form of responsiveness that was notable during recording sessions) as here we only examined correct responses. We investigated this possibility at a future stage in the analyses.

The results shown in Figure 3 regarding the time-course of task-modulations (Figs. 3A&B) prompted us to next use a peri-saccade analysis window to quantify in which ways putamen neurons were modulated by saccade context in the behavioral paradigm. We chose a 100-msec window centered on saccade onset to achieve this goal. Here, we classified significant saccade neurons based on a two-way ANOVA (p<0.01) with factors rule (pro, anti) and saccade direction (contraversive, ipsiversive). We also determined whether the difference between the activity in the peri-saccade window and the baseline window were significantly different (paired t-test, p<0.01).

This analysis demonstrated that as many as 103 (42%) neurons displayed a significant modulation by rule (n = 4) saccade direction (n = 11), rule and saccade direction (n = 3), interaction of rule and saccade direction (n = 18) or a significant overall change in activity during the peri-saccade period in comparison to the baseline period (n = 67). We refer to the latter category of cells as “generally responsive” whereas all other categories are considered to be “task-modulated”. Note, however, that these numbers were not the final values that we include in forthcoming results summarized in table and figure format.

**Figure 4:**
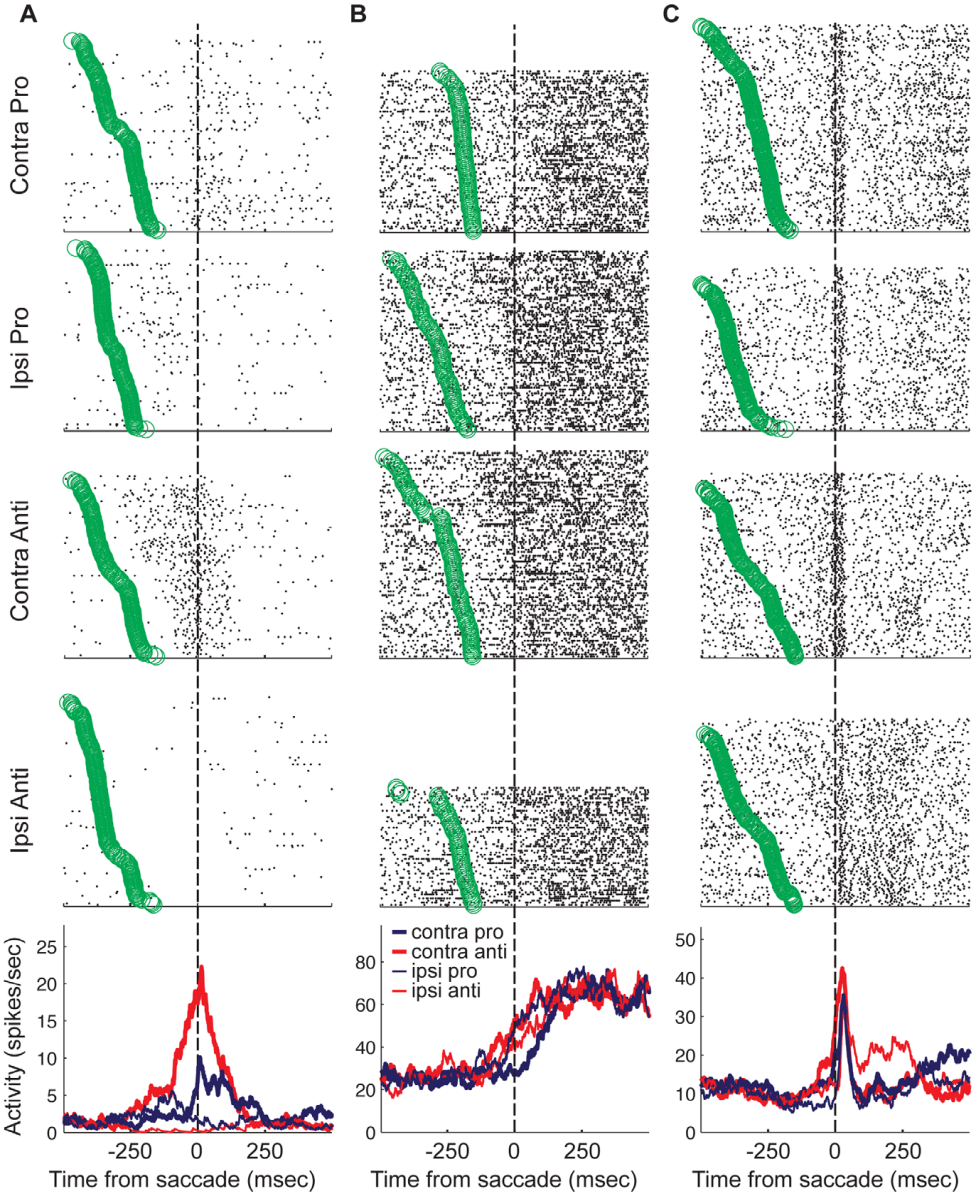
Task-modulated putamen neurons. In each of A, B and C, activity was plotted for a single neuron that showed task-modulated activity during the peri-saccade period. For each neuron, rasters are plotted separately for each task condition. The circles that appear with the rasters represent the time at which the peripheral stimulus was presented in that particular trial. The peri-event spike time histograms (PSTHs) are plotted in the lower panel. Each neuron displayed a unique pattern of context-dependent peri-saccade activity (see results).

Figure 4 shows the activity of three single neurons that each exhibited peri-saccade activity, each showing a combination of task rule- and saccade direction-modulation. In these examples, task modulation appears just prior to the saccade and increases at saccade onset. The neuron plotted in Figure 4A shows low-level baseline activity. For contraversive pro-saccades, a transient increase was observed at saccade onset lasting roughly 250 msec. For ipsiversive pro-saccades, this same neuron showed a slight increase from baseline during a 200-msec period beginning approximately 200 msec prior to saccade onset. For ipsiversive anti-saccades, it appears that neural activity was suppressed before the saccade and during the epoch that we analyzed. The most robust response displayed by this neuron was for contraversive anti-saccades. There was an increase in activity from baseline beginning at a similar point in the trial as the response preceding ipsiversive pro-saccades. However, this increase evolved into a relatively large burst, which peaked just after saccade onset for this task condition. It appears that this neuron responded to an ipsilaterally presented stimulus, with reduced activity during the saccade period for the pro-saccade rule, but a robust increase in the same epoch for the anti-saccade rule.

The activity pattern is quite different for the neuron in Figure 4B. This neuron had a higher tonic level of baseline activity. The activity increased to a common level following saccades in all four task conditions, however, differences are evident during the saccade epoch. The increase in activity began earliest for contraversive anti-saccades, and contraversive pro-saccades gave rise to the largest saccade-period latency. Thus, the largest contrast in activity for this neuron was between contraversive anti-saccades and pro-saccades, which required saccades to be directed toward the same location, but differed in the stimulus-response contingencies.

Finally, the third example is shown in Figure 4C. This neuron was tonically active, but at a lower level compared to that shown in Figure 4B. The neuron showed a phasic burst of activity beginning before, but peaking immediately after saccade onset. Again, context-dependent activity is evident during the saccade epoch. The largest pre-saccade increase occurred for contraversive anti-saccades. In this condition, the activity also rose to a larger peak earlier than for the other conditions. For the other three conditions, the activity began to rise closer to the time of saccade onset, and the peak activity was lower. For ipsiversive anti-saccades, the activity was sustained briefly after the peak was reached. For this neuron, this was the only condition that was associated with a post-saccade sustained activity level.

**Figure 5:**
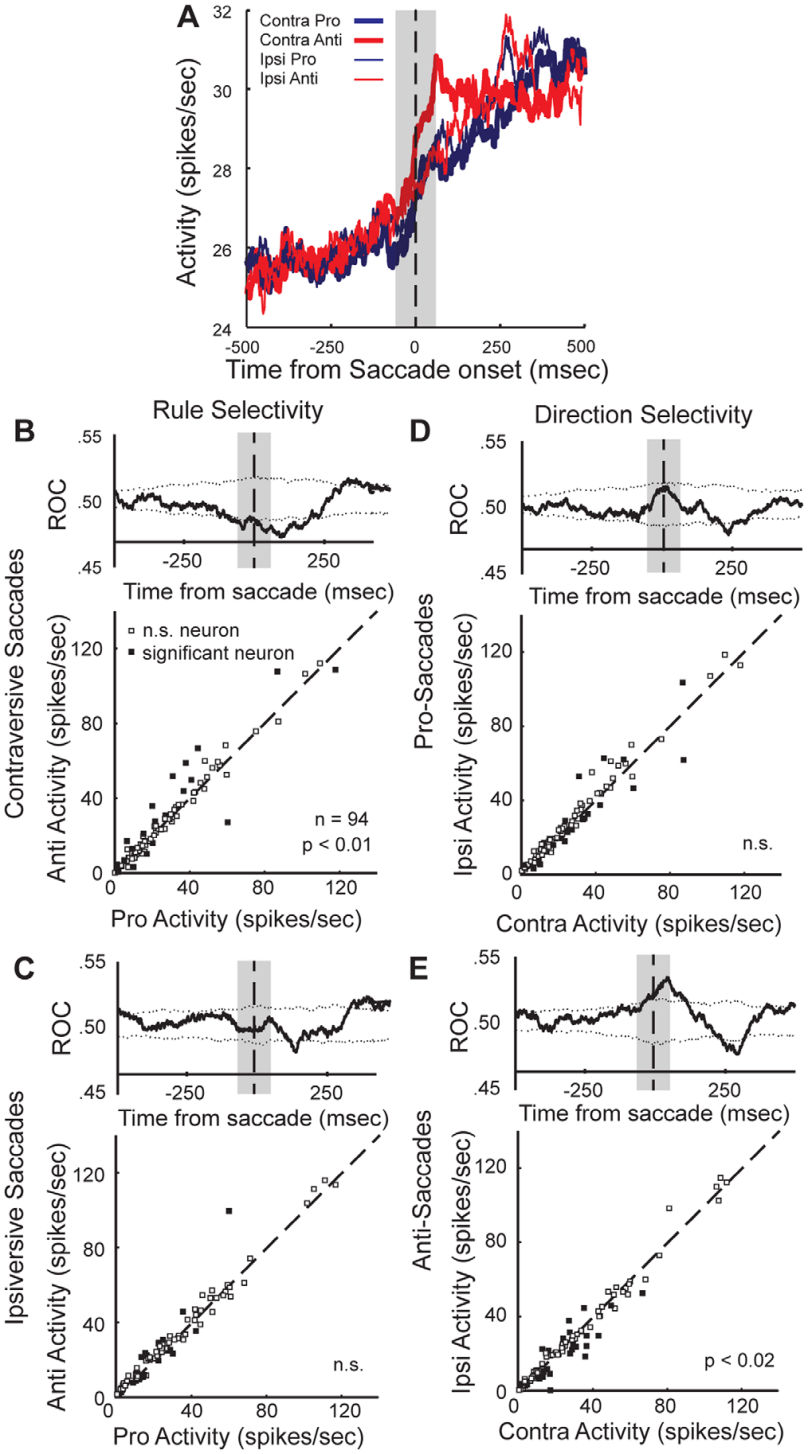
The population of saccade neurons shows task-selectivity during the peri-saccade period. A. Mean population activity of saccade neurons (p<0.01, two-way ANOVA) is plotted, aligned on saccade onset. The grey box indicates the analysis window. B&C. Activity of individual neurons is plotted for pro- versus anti-saccade trials for contraversively (B) and ipsiversivally (C) directed saccades (rule selectivity). A Wilcoxon ranked-sum test was performed for each neuron during the analysis window. For each comparison, the time-course of the mean ROC area was plotted above each scatterplot. D&E. Activity of individual neurons for contraversive versus ipsiversive saccades is plotted, separately for pro-saccades (D) and anti-saccades (E) (saccade direction selectivity). The activity was compared statistically using a Wilcoxon ranked-sum test, and the ROC time-course was plotted as in B&C.

For a closer examination of the nature of contextual saccade modulations within the activity of the population of neurons that were modulated during the saccade epoch, we separated trials by condition and plotted the mean population activity for each (Fig. 5A). Here, it is evident that the peri-saccade increase in the population activity was most robust for contraversively directed anti-saccades, compared to pro-saccades (p<0.01, Wilcoxon signed-rank test) and compared to ipsiversive anti-saccades (p<0.02, Wilcoxon signed-rank test). In addition, we examined the activity of individual neurons. We separated trials based on the saccade direction and plotted the mean activity in the peri-saccade analysis window for pro- saccades versus that for anti-saccades. We also and tested the activity of the individual neurons for differences between trials associated with a saccade in the same direction but selected under a different rule. This demonstrated that single neurons showed task differences for contraversive saccades (Fig. 5B lower panel, n = 19 (20.2%), p<0.05 Wilcoxon ranked-sum test, Table 1). Further, the time-course of significant differences in the two distributions (activity for pro-rule versus activity for anti-rule trials) was determined by plotting the mean receiver operating characteristic (ROC) area of the population of saccade neurons (Fig. 5B, upper panel). For contraversive saccades, the population discriminated between the two tasks during the saccade epoch and this difference persisted into the post-saccade period. For ipsiversive saccades, there was no overall effect of task-rule on the average population activity during the peri-saccade period (Fig. 5C, p>0.05, Wilcoxon signed-rank test). Although the population showed no statistical difference in mean activity between trials with different rules for ipsiversive saccades, a comparable percentage of the single neurons showed task rule-related differences for ipsiversive saccades as that observed for contraversive saccades (Fig. 5C, n = 23 (24.5%), p<0.05 Wilcoxon ranked-sum test, Table 1). This implies that the lack of overall population modulation by ipsiversive saccades because a similar proportion (of single neurons) was modulated for each task rule and further, the rule effect within each individual neuron was not as robust as that which was observed for contraversive saccades.

**Table 1:** Specific task preferences of individual neurons within the population of saccade neurons (neurons tested, p<0.05, Wilcoxon ranked-sum test for all categories of selectivity other than non-specific, p<0.01 grand mean of ANOVA compared to baseline).

Task Selectivity	#Neurons	%Modulated	%Tested	%Population
Contra rule	15	39	16	6
Ipsi rule	20	51	21	8
General rule	4	10	4	2
Total rule	39	100	41	16
Pro direction	12	35	13	5
Anti direction	11	31	11	4
General direction	12	35	13	5
Total direction	35	100	37	14
Generally responsive	41	100	44	17

The second main effect in the saccade-epoch ANOVA was saccade-direction. There was a similar percentage of neurons that were modulated by saccade direction, as that for neurons modulated by task rule (Fig. 3B). To investigate the nature of direction modulation in putamen neuron activity, we separated trials based on the rule that instructed the saccade. Here we compared population activity between trials that required a different saccade direction but the saccades were guided using the same task rule (Fig. 5D&E). Here, it was evident that as a population, the putamen neurons discriminated between saccade-direction in trials having an anti-saccade rule, but not pro-saccade rule, during the peri-saccade epoch. This was significant in the mean population activity (Fig. 5A, p<0.02, Wilcoxon signed-rank test), and the ROC time-course revealed that that the difference occurred at saccade onset and persisted briefly into the post-saccade period (Fig. 5E, upper panel). The activity of single neurons also showed direction modulation on anti-saccade trials (Fig. 5E, n = 24 (25.5%), p<0.05, Wilcoxon ranked-sum test). Despite that there was no significant difference in the population for pro-saccade trials generated in opposite directions (Fig. 5D, upper panel), the same percentage of single neurons showed significant differences in activity between pro-saccade responses with different saccade directions as was observed for anti-saccade trials (Fig. 5D, lower panel n = 24 (25.5%), p<0.05, Wilcoxon ranked-sum test, Table 1). The differences were not quite as robust for pro-saccade compared to those for anti-saccade trials.

### Putamen Neurons are not Modulated by Saccadic Response-switches

We used a blocked task-switching paradigm because previous reports have implicated the putamen in response-switching [27], [28]. However, we were unable to find any evidence that neurons in the caudal putamen neurons play a role in saccadic response-switching. A three-way ANOVA (p<0.01) was used to examine the responsiveness of putamen saccade neurons on switch trials (i.e., the first trial in a new block wherein the previous rule had to be discarded and task-set reconfiguration was required), with main factors rule (pro, anti), saccade direction (contraversive, ipsiversive) and switch performance (correct, error). In this analysis, the number of modulated neurons was not different than what would be expected by chance. We next submitted neural activity to an additional ANOVA. In this next case we examined all correct trials and used the main factors of rule (pro, anti), saccade direction (contraversive, ipsiversive) and trial type (switch, repeat). This analysis again failed to indicate that putamen neurons are modulated by rule-switches. Therefore, we did not pursue switch-related analyses further and instead, we conclude that this striatal region, which appears to correspond to an oculomotor zone within the caudal putamen, is not directly involved in over-riding a prepared saccade in the face of an unexpected change of context. This was not entirely surprising, given the previously demonstrated importance of the rostral striatum and subthalamic nucleus in preparing for saccadic response-switching in similar blocked rule-switch tasks [26], [36], [41] and also the lack of preparatory saccade-related modulations in this population.

### Saccade Neuron Population Shows Enhanced Peri-saccade Task-modulation on Controlled Mode Trials Associated with Ipsilateral Stimulus Presentation

**Figure 6:**
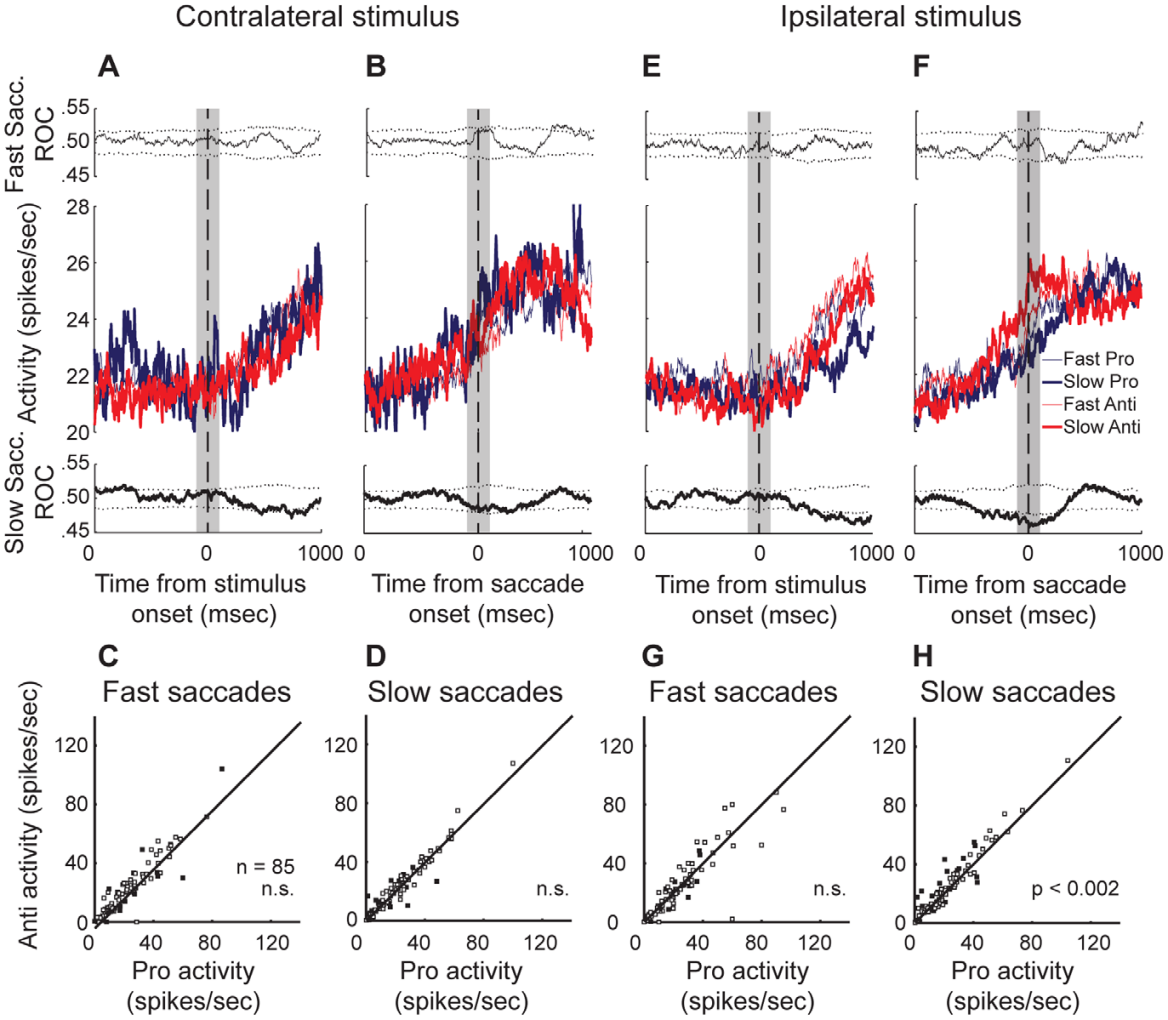
Saccade neuron population shows enhanced rule selectivity for slow saccade responses. A–D. Rule selectivity on fast mode compared to slow mode trials for saccades made in response to a contralateral stimulus. Mean population activity for saccade neurons aligned to stimulus presentation (A) and to saccade onset (B). Upper panel: Time-course of mean ROC value for trials having fast saccade responses. Lower panel: Time-course of mean ROC value for trials having slow saccade responses. C&D. Activity of individual neurons for pro- versus anti-saccades is plotted for fast saccades (C) and slow saccades (D). Filled squares represent neurons that had a significant rule difference (p<0.05, Wilcoxon ranked-sum test) during the analysis window (gray box). E–H. Rule selectivity on fast mode compared to slow mode trials for saccades made in response to an ipsilateral stimulus. Results are plotted as in A–D, but for trials having a stimulus presented in the opposite visual hemifield.

The caudal putamen has been implicated in the capacity to utilize strong stimulus-response associations in diverse mammalian species [29], [30], [31], [32], [33], [34], [35], [36]. Therefore, in this next analysis of saccade neuron activity, we compared activity for trials in which the stimulus was presented to the same visual hemifield. We next separated trials into categories based on the chosen preparatory mode (see Materials and Methods) and then tested the population of saccade neurons for rule-modulation, separately within each mode of response preparation. Figure 6A&B show the mean population activity for trials having a contralaterally presented stimulus (i.e., contraversive pro-saccades and ipsiversive anti-saccades), plotted separately for trials prepared using different speed-accuracy prioritization. The activity is aligned to the onset of the peripheral stimulus in Figure 6A, and the onset of saccade in Figure 6B. The mean ROC area curves for fast (short SRT) saccades are plotted above the population waveforms, while those for slow saccades are plotted below the population waveforms. In the population, there were no statistically significant rule differences for trials performed with either an automatic (short SRT) or controlled (longer SRT) processing mode (p>0.05, Wilcoxon signed-rank test). We also plotted the activity of individual neurons on pro-saccade versus anti-saccade trials having fast responses (Fig. 6C) and slow responses (Fig. 6D). Although there were no rule differences when the neurons were combined as a population, some individual neurons did express rule-modulation, for fast trials (n = 14 (17%), p<0.05 Wilcoxon ranked sum test) and some for slow trials (n = 14 (17%), p<0.05 Wilcoxon ranked sum test). Half of these neurons were included in both groups, while the other half were only rule-modulated during saccades prepared using one mode or the other, which suggests mode-dependence.

**Figure 7:**
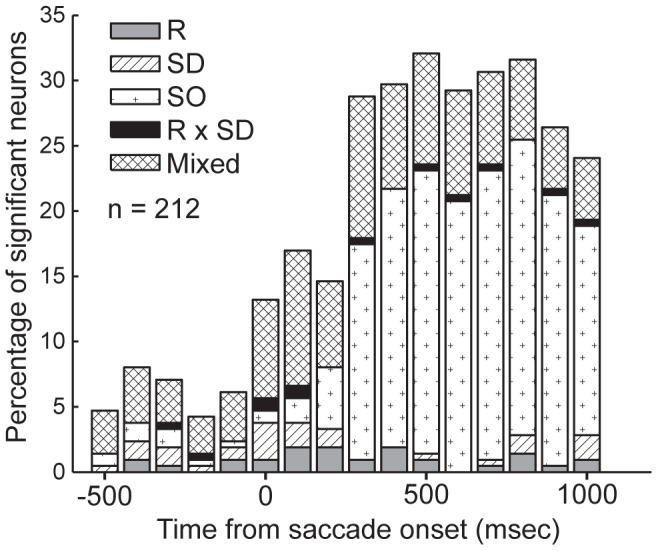
Time-course of ANOVA-identified percentage of task- and outcome- modulated neurons. To explore the time-course of outcome-related, in addition to task-related modulations in the population of putamen neurons, a three-way ANOVA with factors rule (R) (pro, anti) saccade direction (SD) (contraversive, ipsiversive) and outcome (SO) (correct, error) was applied to putamen single neuron activity in 100 msec-wide analysis bins around and extending beyond the peri-saccade period.

We next examined the neural activity in the same way for trials having an ipsilaterally presented stimulus (i.e., ipsiversive pro-saccades and contraversive anti-saccades). Here, we observed a significant rule-modulation in the population of saccade neurons for slow and controlled, but not fast preparatory mode trials (p<0.002, Wilcoxon signed rank test). The mean population activity for saccades having an ipsilateral stimulus, for both fast and slow saccades, is plotted and aligned to stimulus presentation (Fig. 6E) and to saccade onset (Fig. 6F). As in Figure 6A, the mean population ROC area time-course is plotted in the upper panel of Figure 6E&F for fast automatic saccades, and the lower panel of these figures shows that for slower controlled saccades. For slow controlled mode trials, when the stimulus was presented ipsilaterally, the population showed a significant increase around the peri-saccade period for anti-saccades compared to pro-saccades. By contrast, the activity on fast automatic mode trials for both rules reached an indistinguishable intermediate level. According to the ROC analysis for slow saccades (Fig. 6F, lower panel), this rule difference occurred approximately 200 msec prior to saccade onset and persisted beyond the 100-msec analysis window. Therefore, putamen neurons were preferentially activated when the ipsilateral stimulus required the volitional generation of a saccade to the contraversive direction – but only on trials in which the monkeys favoured accuracy over speed, and awaited the instruction cue to retrieve and implement the task-set. This kind of preference was also observed in the activity of the individual neurons. For automatic saccades, 10 neurons (12%, p<0.05 Wilcoxon signed-rank test, Fig. 7G) showed a rule preference and for slower controlled saccades, 19 neurons (22%, p<0.05 Wilcoxon signed-rank test, Fig. 7H) showed a rule preference. Here, even fewer neurons (n = 4) were modulated by the rule during both fast and slow trials, therefore in this analysis the rule-modulation expressed by these neurons was largely mode-dependent. This finding suggests that the rule-modulation observed in the population was in fact dependent upon the speed-accuracy tradeoff strategy used to apply the rule in each trial.

### Putamen Neurons Discriminate Correct and Rewarded Trials from Erroneous Trials

As described in the methods section, it was evident during the recording sessions that many of the putamen neurons appeared to discriminate correctly performed from erroneously performed trials immediately after the response was executed. We used the same method to probe the time-course of trial outcome modulations that was used to investigate that for saccade-modulated neurons. Figure 7 displays the results of this analysis. The three-way ANOVA with factors of task rule (pro, anti), saccade direction (ipsiversive, contraversive) and trial outcome (correct, error), which was conducted using neural activity in 100-msec test epochs, demonstrated that beginning 200 msec following saccade onset, approximately one fifth of the neuron population was significantly modulated by at least one factor, and by 300 msec following the saccade, this proportion increased to nearly one third. This modulation of the population persisted for longer than 1000 msec following saccade onset. This analysis also shows that when trial outcome is taken into account, a portion of the oculomotor selectivities (for rule, saccade direction, or stimulus location) may in fact have interacted with the trial outcome (the number of neurons that showed dual individual main factor modulations or between factor interactions are represented in the “mixed” category, compare results shown in Fig. 3 to those shown in Fig. 7). In the following analyses, we compare correctly executed saccade trials with those trials in which the erroneous saccade was produced in the same direction, so that the oculomotor response was similar but generated under different rule and outcome conditions (e.g., we compared correct contraversive pro-saccades with erroneous saccades generated toward the contralateral target during anti-saccade blocks).

**Figure 8:**
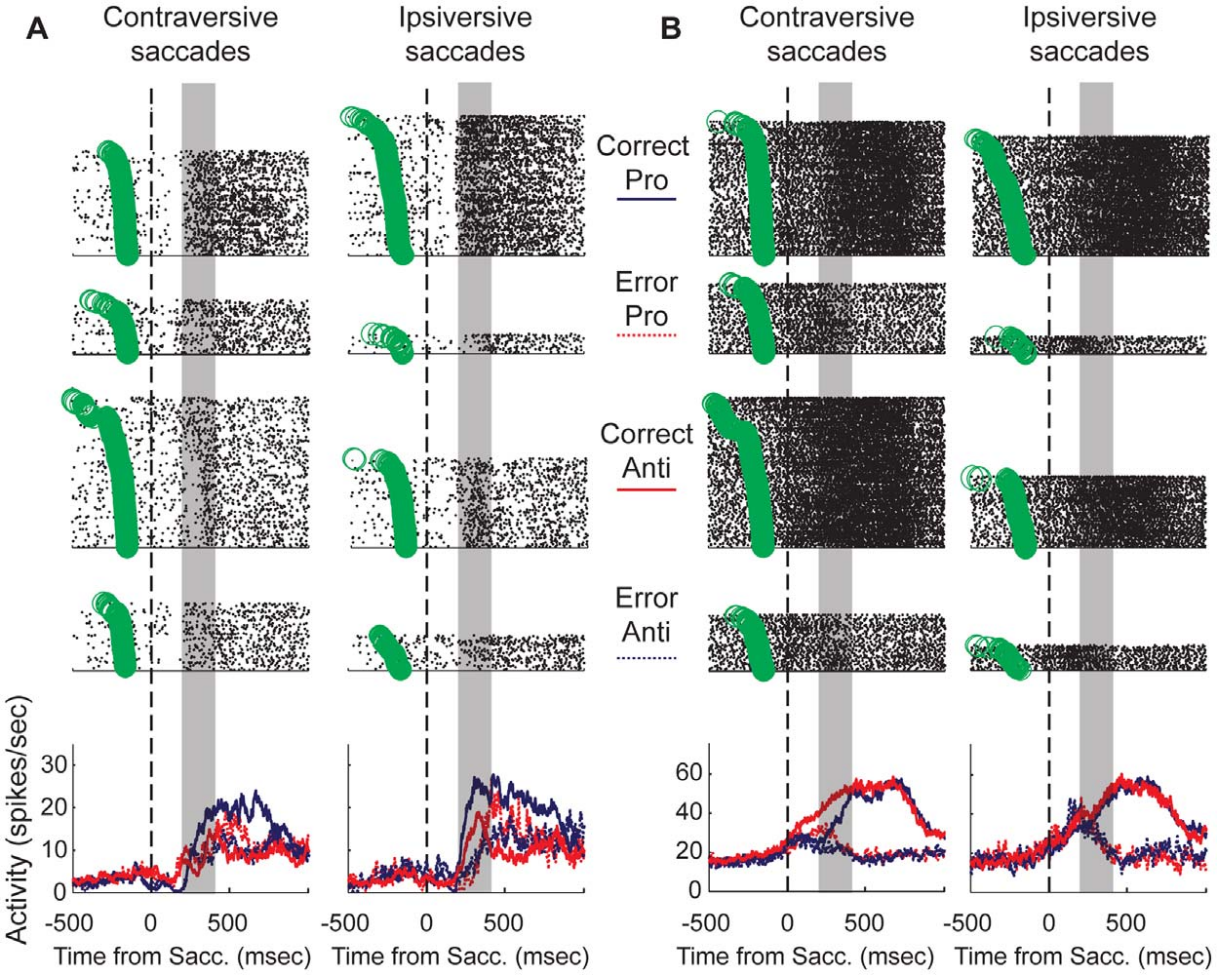
Single putamen neurons show selectivity for trial outcome. A&B each show an example of a neuron that showed task- and outcome-dependent activity just after the saccade. The rasters are plotted separately for each condition above the overlaid PSTHs. The green circles represent the onset of the peripheral stimulus. Each neuron shows a unique pattern of task-specific outcome selectivity (see results).

In Figure 8, we have plotted activity of single neurons that showed combinations of task and outcome modulation. It is evident here that these neurons did not simply discriminate all correct trials from error trials. The fact that the activity interacted between the factors of outcome, task rule and saccade direction indicates that this part of the striatum could contribute to the maintenance of immediate context for goal-directed saccade behavior. The neuron plotted in Figure 8A showed a low level of baseline activity. Approximately 250 msec following saccade onset, the activity of this neuron reflected the behavioral outcome. The most robust activity increase was observed for correctly performed ipsiversive pro-saccades. There appeared to be a unique pattern of post-saccade activity expressed by this neuron, depending on the rule and the direction of the saccade.

In Figure 8B, we have plotted the activity from another neuron that was modulated by the outcome of the just-executed saccade. This neuron had a higher level of baseline activity prior to saccade onset in comparison to the neuron plotted in Figure 8A. It showed a sustained increase in activity that began just before saccade onset for correctly performed pro- and anti-saccade trials. For erroneously performed trials, this increase was initiated but truncated approximately 200 msec after the saccade. For correctly executed responses generated in the contraversive direction, the activity discriminated between the two task rules. For anti-saccade trials, the increase occurred at a steady rate, while for pro-saccade trials, the activity was delayed until approximately 200 msec following the saccade, when it continued to rise to a similar level as that for correct anti-saccade trials.

**Figure 9:**
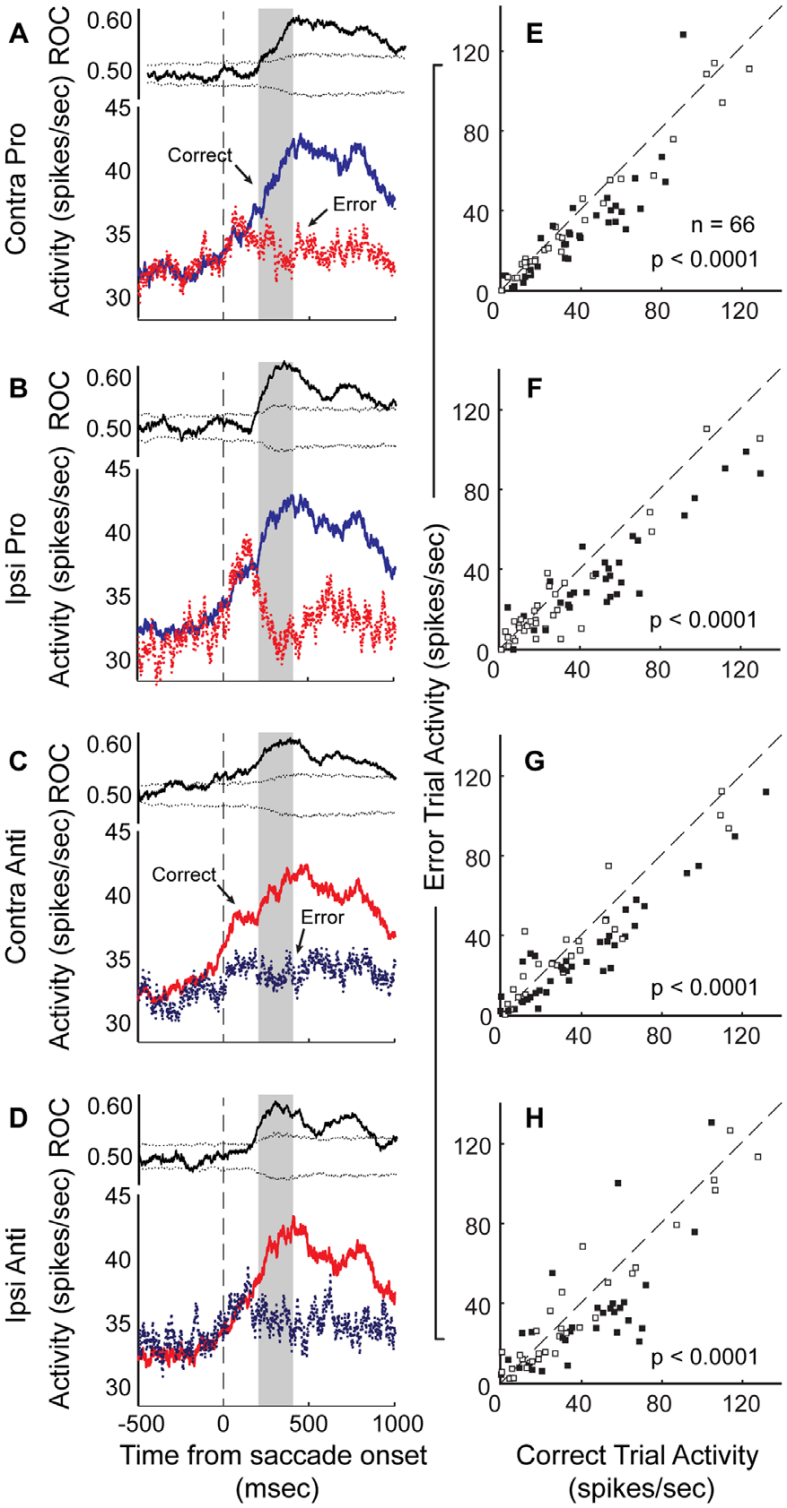
Activity of putamen neuron population is modulated by trial outcome. A–D. For each task condition, the mean PSTH for correctly executed trials and erroneous trials is plotted (bottom panels), aligned to saccade onset. We compared correct and error responses in which the saccade direction was the same (i.e., activity for correct contraversive pro-saccades was compared to that on error trials for the ipsiversive pro-saccade condition). In A–D, the upper panel shows the mean ROC area for the distributions of neural activity in the two conditions. In E–H, the mean activity during the post-saccade period (200 msec following saccade onset for 200 msec) was plotted for correct trials versus that for error trials.

To quantify the population activity related to trial outcome in the population, we selected a 200-msec-wide test window beginning 200 msec after saccade onset. This decision was motivated by the results plotted in Figure 7. We used a three-way ANOVA with factors of task rule (pro, anti), saccade direction (contraversive, ipsiversive) and trial outcome (correct, error) on the entire population. Here, the activity of 66 (31.1%), out of the 212 neurons for which a sufficient number (n = 5) of error trials in each task condition were performed, showed a significant main or interaction effect. Figures 9A–D show the population PSTHs and mean ROC area time-courses for correct and erroneous trials, plotted separately for each task condition. For all conditions, the population of neurons discriminated between correct and error trials after saccade onset (p<0.0001, Wilcoxon signed-rank test), but showed similar rise in activity (relative to baseline) prior to the saccade regardless of whether or not it was produced toward the correct location. For contraversive anti-saccades, the ROC area curve indicates that the population discriminated performance of the forthcoming response immediately before the saccade was initiated. In addition, a large proportion of the individual neurons that were included in this analysis showed significant differences in activity after correct, compared to erroneous trials (Figs 9E–H, contra pro: n = 38 (66%), ipsi pro: n = 33 (55%), contra anti: n = 35 (53%), ipsi anti: n = 35 (53%), p<0.05, Wilcoxon ranked-sum test, Table 2).

**Table 2:** Specific preferences of individual neurons within the population of outcome-modulated neurons (neurons tested, p<0.05, Wilcoxon ranked-sum test).

Outcome Selectivity	#Neurons	%Modulated	%Tested	%Population
Contra Pro	10	16	15	4
Ipsi Pro	2	3	3	1
All Pro	3	5	5	1
Contra Anti	6	10	9	2
Ipsi Anti	4	7	6	2
All Anti	6	8	8	2
Mixed Rule	20	44	38	10
All Conditions	13	13	12	3
Total	64	100	97	26

**Figure 10:**
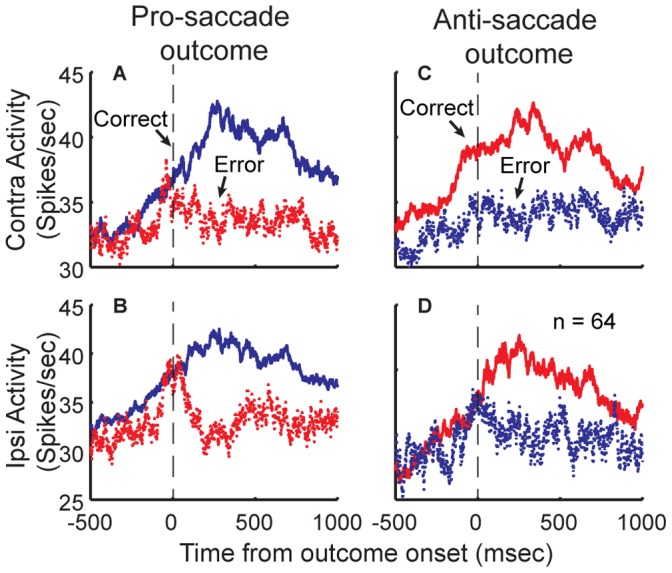
Outcome-modulated population activity aligned to the onset of outcome. A–D. For each task condition, we re-plotted the mean population activity (as shown in Figure 9) of outcome-modulated neurons aligned with the outcome.

To demonstrate the relation of this outcome-dependent modulation to the timing of the reward, we also plotted this same population activity but aligned to the outcome rather than the saccade (Fig. 10). This figure further supports the notion that this neuronal signal might be related to the anticipation and obtainment of a positive outcome, since the activity increases before the response and reward, but diverges on erroneous trials at the time of the outcome.

### Overlap of Context Modulation-dependent in the Population of Putamen Neurons

We have conducted three separate analyses on the activity of this population of putamen neurons. These analyses have indicated that putamen neurons are modulated during the saccade period, that the mode of saccade preparation might influence task modulation in the population of putamen neurons, and that putamen neurons are also modulated by the immediate trial outcome. However, the effect of preparatory mode on single neuron activity has not not been directly tested [42]. To examine the dependence of preparatory mode upon task modulation during the peri-saccade period, we performed a three-way ANOVA (p<0.01) with factors task rule (pro, anti), saccade direction (ipsiversive, contraversive) and preparatory mode (fast internally-cued, slow externally-cued). A neuron was considered to have mode-dependent task modulation if it had a significant rule x mode or direction x mode (or rule x direction x mode) interaction. Similarly, we used the results of the previous three-way ANOVA (p<0.01) for the post-saccade epoch dependence of neural activity on the trial outcome and task condition variables to classify the neurons that were significantly modulated in this later epoch.

**Figure 11:**
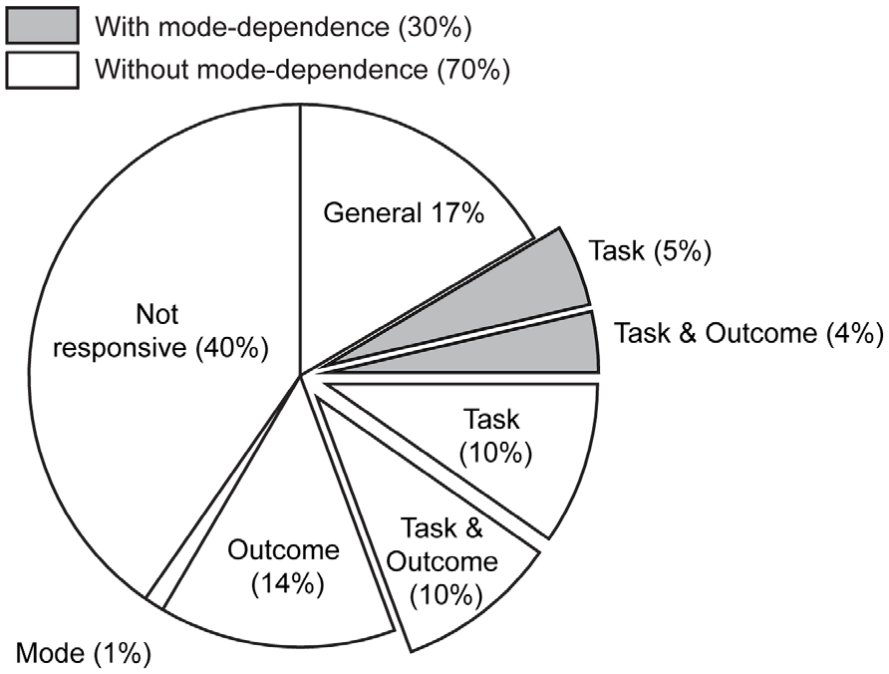
Summary of context selectivity in the population of putamen neurons. The full chart represents 100% of the putamen neurons that we recorded. General responsiveness refers to the neurons that only showed a significant difference in activity during the peri-saccade epoch when compared from the baseline period. Task modulated refers to neurons that displayed task, saccade direction or mixed selectivity during the peri-saccade period. The “exploded” slices indicate any combination of neuron selectivity that involved task-selectivity. The percentages of task-modulated neurons, for which the expression of rule-selectivity was dependent upon the preparatory mode, are represented in the shaded slices. Outcome modulated refers to neurons showing different activity levels for correct and error trials during the post-saccade epoch. The remaining classifications indicate that the neurons expressed significant selectivity for a combination of these factors. The specific characteristics of selectivity for neurons in these categories were not uniform (Tables 1&2). Significant task, mode and outcome-dependent activity modulations were assessed by two three-way ANOVAs during two saccade-aligned epochs (see results).

To summarize the quantity of recorded putamen neurons that exhibited the various forms of context-dependent modulation, and the degree of overlap within single neurons for saccade, task and outcome modulation, we displayed the values of these final analyses as percentage of total recorded neurons (n = 245, Fig. 11). Here, the percentages of single neurons having either statistically significant differences in activity relative to baseline during the peri-saccade epoch (general responsiveness), a significant main effect or interaction effect according to a three-way ANOVA (p<0.01), or no responsiveness, are shown. Overall, 60% of the putamen neurons that we recorded were significantly modulated in some way during performance of the SOT. Without having any specific contextual modulation, 17% (n = 41) of all recorded neurons showed a general, non-specific change from baseline activity during the saccade period. Outcome and task exerted an influence on the activity of a roughly equivalent percentage of putamen neurons. With all combinations of factors taken into account, modulation for trial outcome after the saccade influenced the activity of 28% (n = 68, or 47% of modulated neurons) of neurons that we recorded. Including all combinations of factors showing task modulation, 28% (n = 69, or 48% of modulated neurons) of recorded neurons were sensitive to the task rule, saccade direction, or a combination of these factors. Because peri-saccade rule-modulation was found to be dependent upon the mode used to prepare the saccade, we plotted the percentage of neurons that were task-modulated (i.e., for rule or saccade direction) regardless of the preparatory mode separately from that for which task-modulation was dependent on the chosen preparatory strategy. Mode-dependency in rule-modulation (30% task-modulated) was not as prevalent as those task-modulated neurons whose modulation was mode-independent (70% task-modulated).

Although it is known that some ventro-medial zones within the “motor striatum” are responsive to orofacial or licking movements, and despite that we did not employ any means to monitor mouth and licking movements, we assert that many of the outcome-modulated neurons are not likely to correspond to such neurons. First, half of the outcome-modulated neurons only showed a main effect of outcome. We cannot rule out that the activity of these neurons was motor-related. However, many of these neurons were spatially intermingled amongst the task-modulated neurons and previous studies have demonstrated clear somatotopic segregations in the striatum both anatomically and functionally [1], [5]. The remaining half of outcome-modulated neurons showed either an interaction with task, mode, or task and mode (n = 31) or outcome modulation in the post-saccade period and also task- or mode-modulation in the peri-saccade period (n = 3). Therefore it is unlikely that outcome-modulated activity of these neurons is related to orofacial movements.

**Figure 12:**
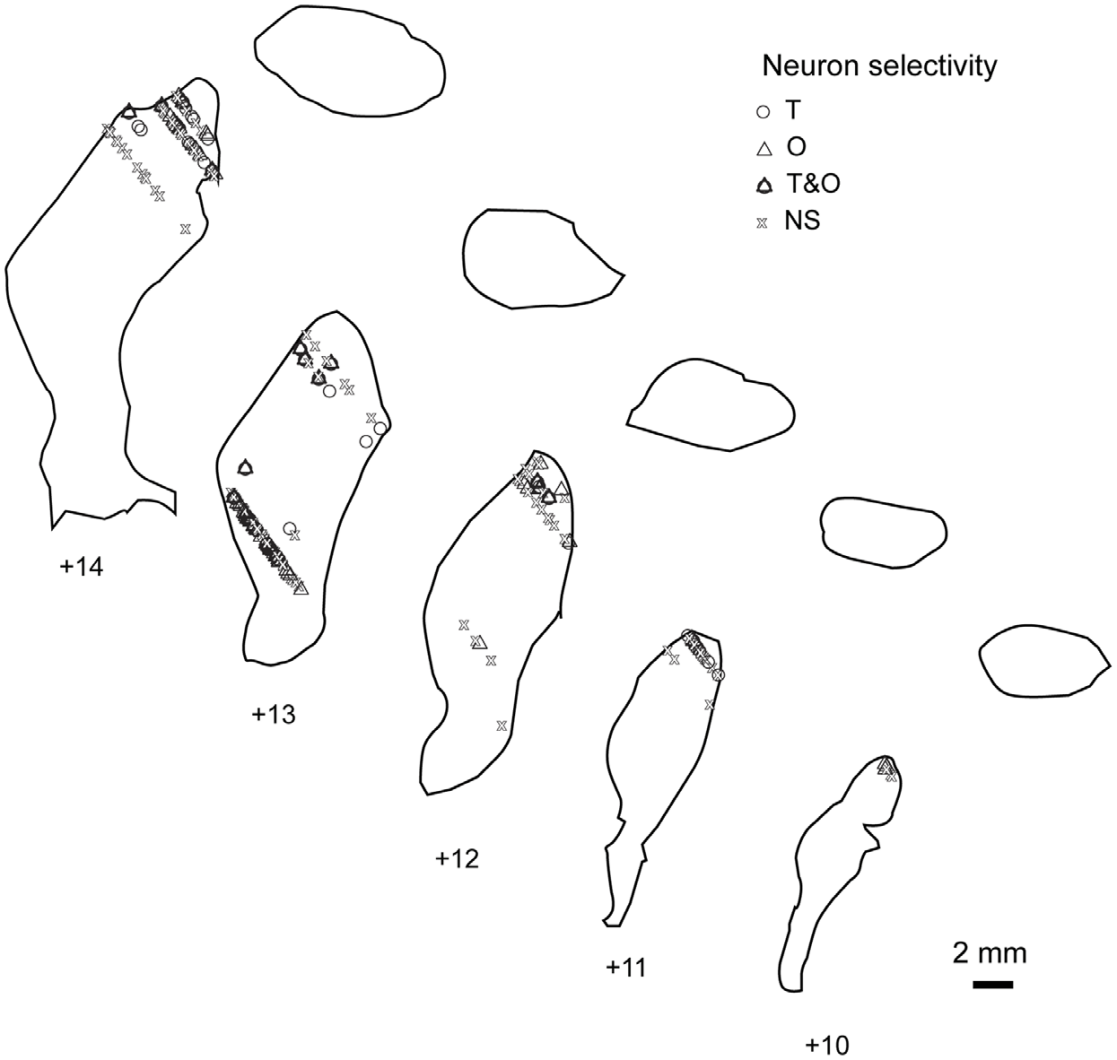
Reconstruction of recording locations including task-related selectivity. Coronal putamen slices were traced by fitting histological boundary drawings (BrainInfo, Seattle, WA) onto the anatomical MRIs for each animal. This figure demonstrates that the recordings likely occurred within the putative “oculomotor zone” of the putamen [21], [22], [23], . Recording locations were plotted onto the combined MRI & traced coronal images. Each symbol indicates a single neuron. The shape of each symbol conveys the kind of task selectivity observed for the plotted neurons. The anterior-posterior coordinates are given with respect to the inter-aural line. The selectivity categories are as described with Figure 11.

In Figure 12, we have plotted the recording location of each neuron in our population. We targeted the dorso-caudal putamen and sampled any well-isolated unit that was encountered as the electrode was passed ventro-medially. The locations of the recording sites mostly fall within the approximate locations of putative terminals from the FEFs and SEFs that have been previously shown in monkeys [21], [22], [23], [24], which is consistent with our observations of saccade-related modulation within the population of neurons.

## Discussion

Here, we recorded extracellular activity from single neurons in the caudal portion of the putamen while monkeys performed short alternating blocks of pro- and anti-saccades. We found that as a population, these neurons expressed a preferential increased activity for contraversive anti-saccades, and that neurons in this region were also modulated by the outcome of saccades in a context-dependent manner. Further, we report that the preparatory strategy used to perform pro- and anti-saccades significantly impacted the expression of task-modulation in the population, specifically for roughly one third of the task-modulated neurons. This data implies that the macaque putamen may contribute to saccade control, which is consistent with functional neuroimaging studies in human subjects [4], [5], [6], [7], [8], [9], [10], [12], [43], [44].

To date, neurophysiological investigations into the function of striatal neurons in saccade control have focused on the caudate nucleus. These studies have helped shape modern models of BG function, which place oculomotor and executive control functions on the cortico-BG loops that pass through the caudate [45], [46], [47]. The motivation to focus on the caudate can be attributed to anatomical studies of cortico-BG loops, which have demonstrated massive innervation to the caudate from the dorsolateral prefrontal cortex, FEF and SEF [1], [21], [22], [23], [24], [48], [49]. However, some of these studies showed that the dorso-caudal putamen also receives inputs from FEF and SEF [21], [22], [23]. Here, we show that approximately half of the neurons that we recorded in this region showed some form of task-related modulation, suggesting that the putamen might play a role in saccade control.

What might the purpose of saccade-modulated activity be in the putamen, in relation to that in the caudate nucleus? Caudate neurons show pre- and peri-saccade activity during saccade tasks that is correlated with SRT, and delay activity when the saccade target must be remembered [50], [51]. When the magnitude of reward associated with each response option is manipulated, which creates high and low value stimuli and responses, caudate activity reflects the animal’s motivational bias [52], [53], [54], [55]. It has been suggested that caudate neurons resolve conflicting visually-triggered activation in both hemispheres for anti-saccade performance, or provide suppression of inappropriate pro-saccades via the indirect pathway [40], [56] and might serve to pre-set the (oculo)-motor system for volitional tasks [46], [57], [58]. Finally, caudate activity reflects the time-course for learning of abstract associations between novel stimuli and saccade responses, and flexibly tracks changing stimulus-response and stimulus-reward contingencies [41], [54], [59], [60], [61], [62], [63]. In sum, caudate neurons are thought to bias saccade selection based upon contextual information contained within their preparatory activity by altering that in the SNpr, which is considered a permissive gate for saccade selection in the superior colliculus [3]. A common finding in these studies, which mainly explored functional activity in the anterior caudate nucleus, was the observation of preparatory modulations in the activity of caudate neurons, which stands in contrast to our observations of predominantly peri- and post-response modulations in the putamen.

Similar to the putamen neurons in this study, caudate neurons also exhibit post-saccade activity [53], [64]. A recent study reported that post-response outcome modulation in the caudate persisted into the next trial and then influenced the magnitude of task-modulation [60]. Caudate neuron activity has been reported to reflect the nature of the just-executed response, or the size of the reward that followed the saccade, while the simultaneous encoding for both kinds of information was rare [64], [65], [66]. In contrast, it was common for putamen neurons in the present study to carry outcome-related information that interacted with task-related information.

Potentially, foveal visual stimulation following a pro-saccade towards the stimulus, which was absent following an anti-saccade away from the stimulus, might have contributed to the observed differences in outcome-related activity between the two trial types. While we cannot fully exclude this possibility, we do not believe that it can solely account for the task and outcome modulations of putamen neurons, because we did not observe putamen neurons that were modulated by the acquisition of the fixation point at the beginning of the trials.

Because the activity patterns of some of the putamen neurons in this study look very similar to published examples of caudate neurons, and further because a subset of post-commissural putamen neurons projects to the SNpr [67], [68], [69], it is possible that at least some of these neurons contributed to saccade generation. During saccade behaviour, putamen neurons might act with the caudate to relieve inhibition in the SC. However, rule-related differences in this population of neurons as a whole appeared in the peri-saccade epoch rather than the preparatory epoch, thus we hesitate to claim that these activations exerted a direct influence on saccade generation. Further, information pertaining to the rule preferentially emerged for controlled, visually-instructed saccades generated in response to an ipsilateral stimulus but not for the same saccadic responses that were prepared using the internally-maintained rule. This rule-modulation that emerged for controlled mode responses could reflect an internal record of task-set retrieval since the two preparatory modes differed in that, for automatically prepared saccades, the task-set was already established at the beginning of the trial. Finally, the predominance of reinforcement-related post-saccade responses further suggests a role in action or performance monitoring rather than saccade preparation.

Together with previous studies, our data indicate that the BG “motor loop”, comprised of the putamen, pallidum, VA/VL and medial frontal cortex [49], [70], might be involved in the monitoring of, and possibly the performance of, goal-directed saccades. The integrity of the putamen appears to be important for the manifestation of the error-related negativity (ERN) [71], a scalp potential that follows performance errors and has been localized to the medial frontal cortex [72], [73], which contains the anterior cingulate cortex (ACC) and SEF, two areas that show outcome-modulation [71], [73], [74], [75], [76]. Further, putamen inactivation impaired reward-history based action selection in monkeys [27]. Lesions to the motor thalamus impaired performance monitoring, post-error adjustments and abolished the ERN [77]. Neurons in the SEF [78] and VA/VL [79] preferentially increase their activity during anti-saccade task performance. Moreover, ACC neurons encode pro- and anti-saccade rules when monkeys shift between blocks of repeating either task based on reward feedback [26], [38], [80] and also can bias performance toward anti-saccades [81]. Therefore, it seems possible that this region of the putamen could monitor goal-directed saccades, and confirm task-set and feedback information through the thalamus toward the ACC and SEF.

This study shows that, like in humans, the macaque putamen is engaged during the performance of saccades. Similar to neurons in the caudate nucleus [66], putamen neurons in previous studies have been reported to show preparatory or anticipatory modulations, and also post-movement and reward period modulations (including the present work), and probably are involved in both action selection and updating context-specific expectations in various contexts. Until now, putamen neuron activity had only been examined during the performance of manual tasks [28], [31], [82], [83], [84], [85]. However, caudate activity has been characterized during the performance of manual and reaching tasks in studies that also examined rostral putamen neurons during the same behavior. Differences between caudate and putamen neuron characteristics were not reported in these studies [52], [62], [63], [64], [82], [83], [85], [86], [87], [88], [89], [90]. Where differences often emerge, is when activity is compared directly between rostral and caudal striatal neurons. Evidence suggests that the rostral striatum is likely involved in the trial and error learning of associations and procedural memories, and in the implementation of top-down control biases in the preparation of forthcoming responses, while the caudal putamen may play a more prominent role in the execution of well-practiced behaviors under pre-established stimulus-response associations [29], [30], [31], [32], [33], [34], [35], [36], [91]. Therefore, our data are consistent with a model of striatal function which considers it a continuum through which information progresses from ventro-medial and rostral regions, in the early planning stages of behavior which require the consideration of internal motivational states and attentional goals, toward the dorsolateral and caudal striatum for the execution and monitoring of that action and its outcome [48]. These sequential intra-trial events may be carried out by the sequential emergence of unique, coherent, large-scale cell assemblies [92], [93], [94], [95], [96], [97], each of which might be functionally inter-connected with one or more specific striatal sub-regions.
